# Field-free molecular orientation by delay- and polarization-optimized two fs pulses

**DOI:** 10.1038/s41598-020-75826-8

**Published:** 2020-11-02

**Authors:** Je Hoi Mun, Dong Eon Kim

**Affiliations:** 1 Department of Physics and Center for Attosecond Science and Technology, POSTECH, Pohang, 37673 South Korea; 2 Max Planck POSTECH/KOREA Research Initiative, Pohang, 37673 South Korea

**Keywords:** Atomic and molecular physics, Chemical physics

## Abstract

Unless the molecular axis is fixed in the laboratory frame, intrinsic structural information of molecules can be averaged out over the various rotational states. The macroscopic directional properties of polar molecules have been controlled by two fs pulses with an optimized delay. In the method, the first one-color laser pulse provokes molecular alignment. Subsequently, the molecular sample is irradiated with the second two-color laser pulse, when the initial even—J states are aligned, and the odd—J states are anti-aligned in the thermal ensemble. The second pulse selectively orients only the aligned even—J states in the same direction, which results in significant enhancement of the net degree of orientation. This paper reports the results of simulations showing that the two-pulse technique can be even more powerful when the second pulse is cross-polarized. This study shows that the alignment and orientation can be very well synchronized temporally because the crossed field does not disturb the preformed alignment modulation significantly, suggesting that the molecules are very well confined in the laboratory frame. This cross-polarization method will serve as a promising technique for studying ultrafast molecular spectroscopy in a molecule-fixed frame.

## Introduction

In recent decades, laser-based techniques for fixing molecules in space have become an important tool for a range of experiments. This is because intrinsic structural information of molecules is averaged out unless the molecular axis is fixed in the laboratory frame^1^. The most widely-adopted technique is a transient alignment achieved by irradiating a fs laser pulse, so-called nonadiabatic (impulsive) alignment technique^2–6^. Because a fs laser pulse is short enough compared to the typical rotational speed of a molecule ($\sim$10 ps), the superposition of excited rotational states is generated after the laser pulse has passed, which gives rise to a periodic repetition of the molecular alignment. The nonadiabatic alignment technique has been used for the diffraction of photoionized electrons^7–9^, and high-harmonic spectroscopy^10–14^ for retrieving the electronic-structure information. The nonadiabatic alignment technique, however, cannot confine the head-versus-tail order of polar molecules. To control the molecular orientation, the laser field must be asymmetric with respect to the plus-minus inversion of laser polarization. The asymmetry can be generated by using combined electrostatic and laser fields^15–19^, collinearly polarized a two-color laser field^20–22^, and a THz laser^23–26^, for examples.

These methods themselves, however, require additional specific efforts to increase the degree of orientation measured from a molecular ensemble. This is because some of the molecules are oriented in one direction, and the others are oriented in the opposite direction in the presence of the orientation potential. Such a limit to the achievable macroscopic orientation has been addressed in many reports. In recent studies^27–30^, rotational quantum-state selection techniques based on inhomogeneous DC-field have been exploited to enhance the net degree of orientation dramatically. As an all-optical method for the state-selective orientation, the utilization of two delay-optimized fs pulses, one with a monochromatic field and the other with a dichromatic (two-color) field, was theoretically proposed^31,32^ and experimentally realized^33–36^. While the state-selection has provided the highest degree of orientation thus far, the all-optical two-pulse technique is more suitable for the high-order harmonic generation experiment because of the available high target density^33–35^.

In previous reports on the orientation of linear molecules^31–34,36^, all the laser fields were polarized linearly in the same directions, and more complex polarizations have not been studied. To orient general polyatomic (asymmetric-top) molecules, it is necessary to confine two molecular axes in the laboratory frame. Therefore, elliptical^30,37^ and crossed polarization^38^ have been employed for the three-dimensional orientation of asymmetric-top molecules. Recently, the interaction Hamiltonian induced by the crossed polarization of a two-color field was derived, and it was demonstrated that it could enhance the degree of orientation of linear molecules in the quasi-adiabatic condition^39^.

In this work, we show that a two-color field with crossed polarization can improve the field-free orientation of linear molecules, by solving the time-dependent Schrödinger equation (TDSE) numerically in the short-pulsed crossed polarization regime for the first time. A series of simulations reveal that the two-pulse orientation technique based on the linear polarization, in fact, deteriorates the degree of alignment of the molecular axis in the laboratory frame, even though the oriented molecular sample has a large up-down asymmetry (high degree of orientation). This is because the preformed molecular alignment by the first one-color pulse can be disturbed significantly by the second pulse. On the other hand, this problem can be circumvented by employing the crossed polarization. In the case of the second pulse with crossed polarization, the molecular orientation is generated without significantly destroying the preformed alignment modulation. As a result, modulations of the alignment and orientation are temporally well synchronized, ensuring the tight angular confinement of the molecular ensemble.

## Theory and simulation

We have used the popular rigid rotor model to describe the rotational dynamics, in which the Hamiltonian of a linear molecule $H_{\text {rot}}$ is given by $B\vec {J}\,^{2}$. Here *B* and $\vec {J}$ are the rotational constant and the angular momentum operator, respectively. With aid of the perturbation theory, the interaction between the molecule and linearly polarized nonresonant two-color laser field $H_\text {int}$ can be given as follows^39^:

1 $$\begin{aligned} H_\text {int}^{\parallel }(t)&= H_\text {align}^{\parallel }(t) + H_\text {orient}^{\parallel }(t), \end{aligned}$$

2 $$\begin{aligned} H_\text {align}^{\parallel }(t)&= -\frac{1}{4}[ E_{\omega }(t)^{2} + E_{2\omega }(t)^{2} ](\alpha _{zz} - \alpha _{xx})\cos ^{2}{\theta }, \end{aligned}$$

3 $$\begin{aligned} H_\text {orient}^{\parallel }(t)&= -\frac{1}{8}E_{\omega }(t)^{2}E_{2\omega }(t)\Big [ 3\beta _{zxx}\cos {\theta } +(\beta _{zzz} - 3\beta _{zxx})\cos ^{3}{\theta }\Big ], \end{aligned}$$

for the parallel polarization of the two wavelengths along the laboratory fixed *Z*-aixs, and

4 $$\begin{aligned} H_\text {int}^{\perp }(t)&= H_\text {align}^{\perp }(t) + H_\text {orient}^{\perp }(t), \end{aligned}$$

5 $$\begin{aligned} H_\text {align}^{\perp }(t)&= -\frac{1}{4}(\alpha _{zz} - \alpha _{xx}) \bigg [ \Big (E_{2\omega }(t)^{2} - \frac{E_{\omega }(t)^{2}}{2} \Big )\cos ^{2}{\theta } + \frac{1}{2} E_{\omega }(t)^{2}\sin ^{2}\theta \cos 2\phi \bigg ], \end{aligned}$$

6 $$\begin{aligned} H_\text {orient}^{\perp }(t)&= -\frac{1}{16} E_{\omega }(t)^{2} E_{2\omega }(t) \bigg [(\beta _{zzz} - \beta _{zxx})\cos {\theta } -(\beta _{zzz} - 3\beta _{zxx})(\cos ^{3}{\theta } - \sin ^{2}{\theta }\cos {2\phi }\cos {\theta }) \bigg ], \end{aligned}$$

for the crossed polariztion with $\omega$ in the *X*-axis and the $2\omega$ in the *Z*-axis. Here, $E_{\omega }(t)$ and $E_{2\omega }(t)$ are the envelopes of the $\omega$- and $2\omega$-laser fields, respectively. $\alpha _{zz}$ and $\alpha _{xx}$ are the polarizability components parallel and perpendicular to the molecule-fixed z-axis (the molecular axis); $\beta _{zzz}$ and $\beta _{zxx}$ are the hyperpolarizability components parallel and perpendicular to the molecular axis. For both polarization configurations, the orientation Hamiltonians are smaller than the alignment ones by approximately two orders of magnitude. The polar and azimuthal angles between the molecule-fixed *z*-axis and the laboratory-fixed *Z*-axis are represented by $\theta$ and $\phi$, respectively. The parallel polarization can align and orient the molecules in the *Z*-axis. Meanwhile, the crossed polarization can orient the molecule in the *Z*-axis, and the alignment direction can be either *Z*- or *X*-axis depending on which field component is stronger than the other one^39^. We have solved the TDSE using the Spherical harmonics as a basis set. While under the parallel field condition the quantum number *M* is preserved, but it is not a good quantum number any-more under the crossed field condition. Since the reflection symmetry in the *ZX*-plane remains, it is possible to separate the total Hamiltonian by two irreducible representations. The symmetry issue and the numerical method for solving the TDSE are provided elsewhere^39,40^. In this work, the OCS molecule has been benchmared as a prototype, which has constants of $B = 0.203~\text {cm}^{-1}$, $\alpha _{zz} - \alpha _{xx} = 27.15~\text {a.u.}$, $\beta _{zzz} = 45.0~\text {a.u.}$ and $\beta _{zxx} = 59.1~\text {a.u.}$^41^. The rotational temperature of the molecular ensemble is set to 3 K. The molecules are irradiated with a two-color laser with the wavelengths of 800 nm and 400 nm. In this condition, the laser frequencies are far from any molecular resonance and much higher than the rotational frequency of molecule.

**Figure 1:**
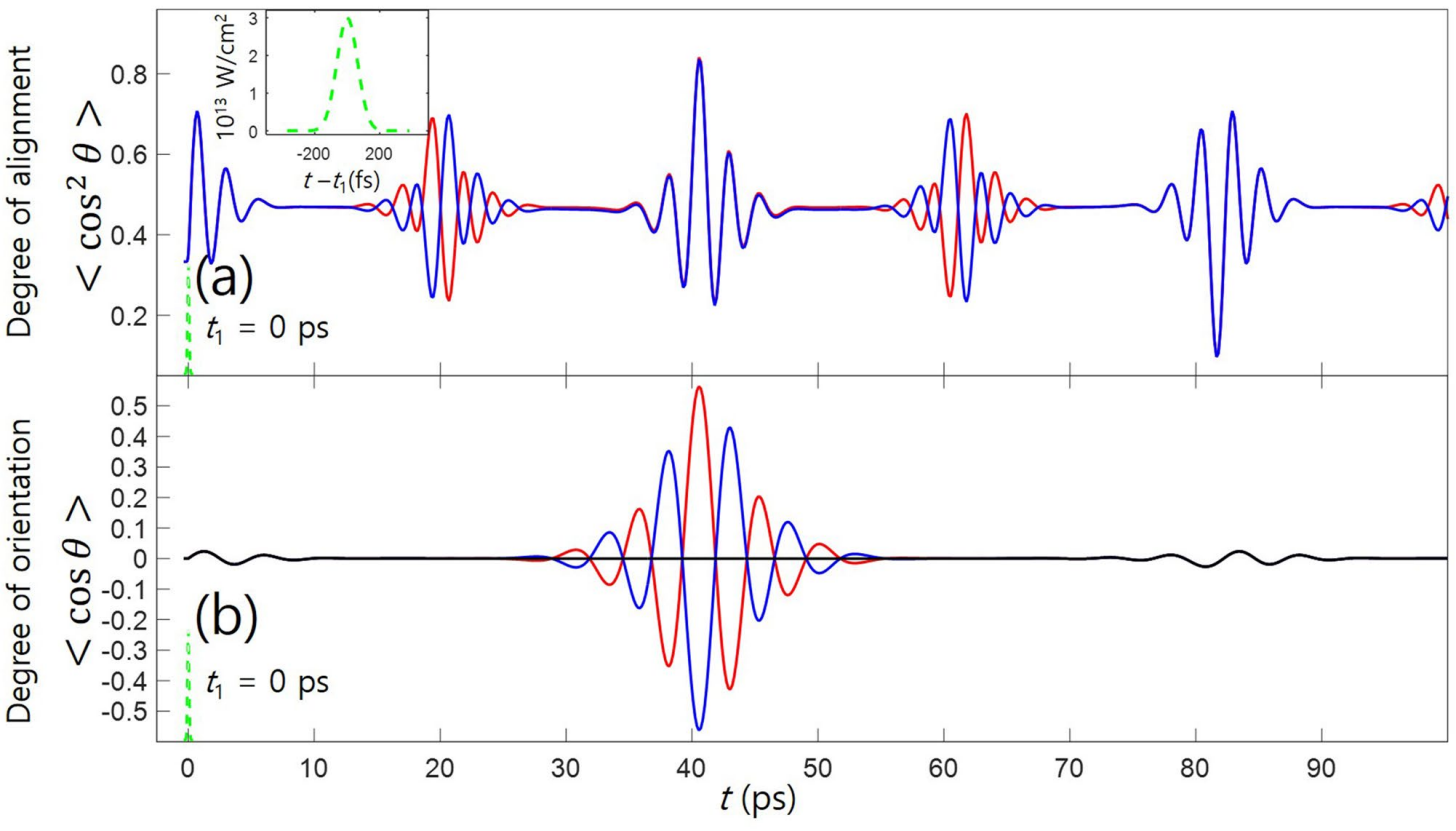
One pulse orientation. Time evolutions of molecular alignment (**a**) and orientation (**b**), after two-color pulse irradiation at $t=t_{1}$. The inset shows the intensity profile of the pulse. The red and blue lines correspond to the even–J and odd–J states, and the black line shows the total contribution of them.

**Figure 2:**
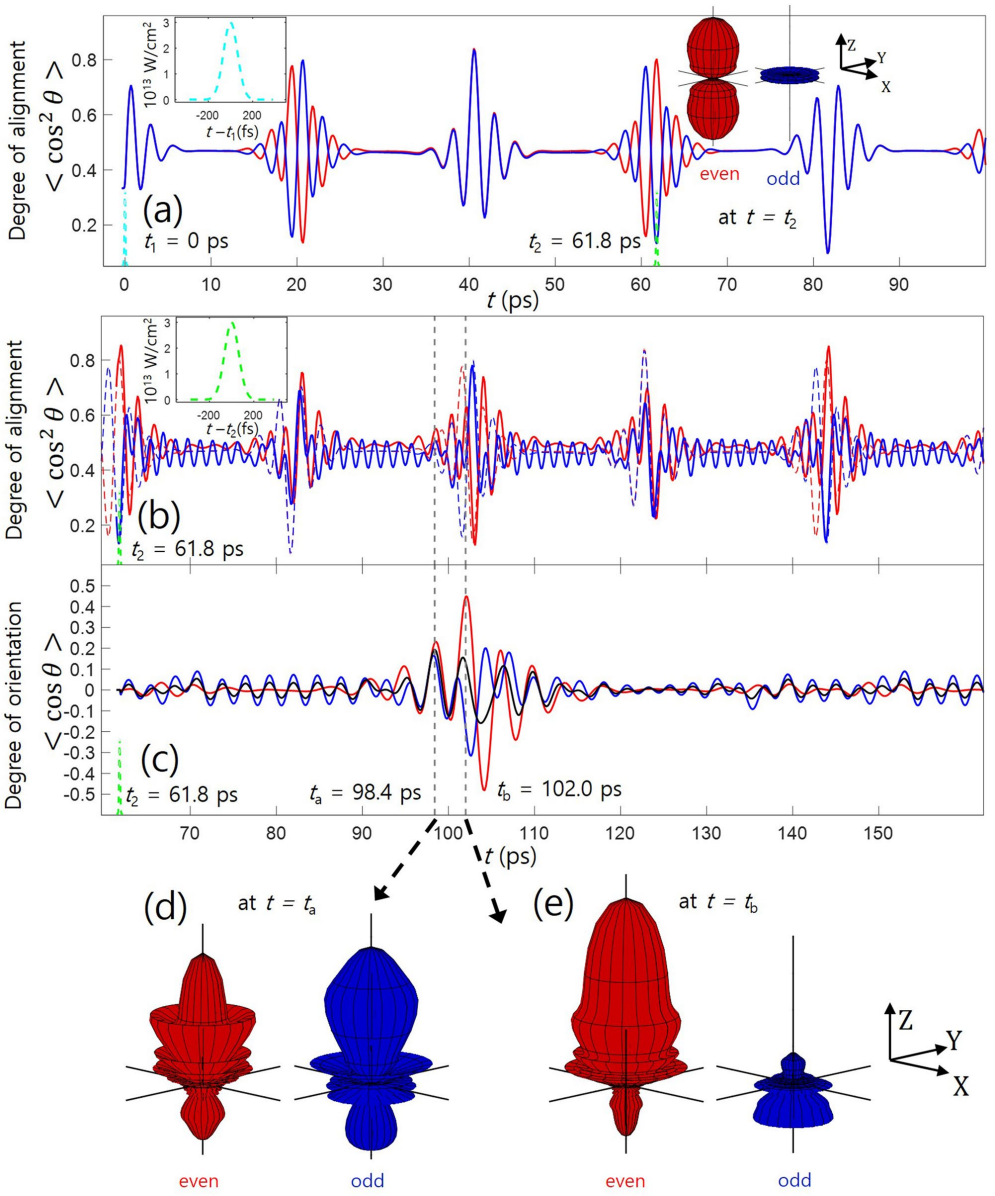
Two pulse orientation with collinear polarization. (**a**) Time evolution of molecular alignment after the first one-color pulse. Time evolutions of alignment (**b**) and orientation (**c**), after the second collinearly-polarized two-color pulse irradiation at $t=t_{2}$. The red and blue lines correspond to the even–J and odd–J states, and the black line shows the total contribution of them. In (**b**), the dashed lines represent the alignment dynamics without the second pulse. The insets in (**a**) and (**b**) show the temporal profiles of the two pulses. In (**a**), the angular distributions of even–J (red, cigar-shaped) and odd–J (blue, disc-shaped) states at $t=t_{2}$ are depicted in the Cartesian coordinates. In (**d**) and (**e**), the angular distributions of even–J (red) and odd–J (blue) states at $t=t_{\text {a}}$ (**d**) and $t=t_{\text {b}}$ (**e) **are depicted.

## Results and discussion

### One pulse orientation (collinearly-polarized two-color pulse)

A two-color pulse collinearly-polarized in the *Z*-axis is irradiated with the molecular ensemble. The peak intensity and the pulse width are $3.0\times 10^{13}~\text {W/cm}^{2}$ and 150 fs by FWHM, respectively. The two wavelength components have the same intensity ratio. After the laser pulse has passed, molecular axis is confined in the *Z*-axis. In Fig. 1a, b, the degrees of alignment ($<\cos ^{2} \theta>$) and orientation ($<\cos \theta>$) achieved by the two-color pulse are shown, respectively. In the alignment, at approximately $t= 20$ ps and $t= 60$ ps, the even–J and odd–J initial states show $\pi -$shifted modulations from each other. At approximately $t= 40$ ps, the highest degree of alignment is observed. At this moment, the even–J and odd–J states are well oriented but in the opposite directions, which results in the net-zero degree of orientation. This has been a traditional problem in molecular orientation

**Figure 3:**
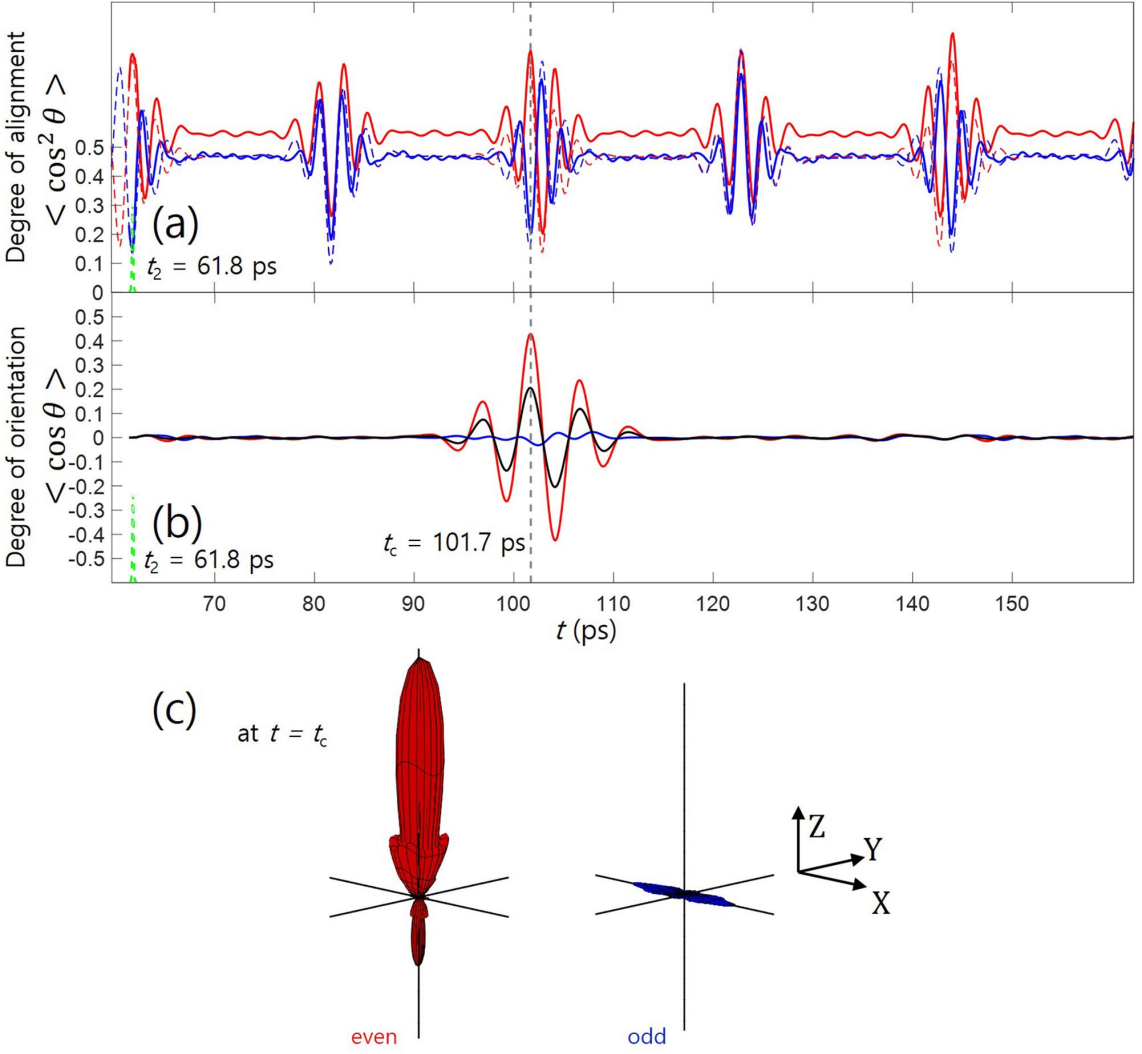
Two pulse orientation with crossed polarization. Time evolutions of alignment (**a**) and orientation (**b**), after the second crossed-polarized two-color pulse. The red and blue lines correspond to the even–J and odd–J states, and the black line shows the total contribution of them. The dashed lines represent the alignment dynamics without the second pulse. In (**c**), the angular distributions of even–J (red) and odd–J (blue) states at $t=t_{\text {c}}$ are depicted.

### Two pulse orientation (one-color pulse and collinearly-polarized two-color pulse)

After irradiation with the first one-color pulse having the peak intensity and pulse width of $3.0\times 10^{13}~\text {W/cm}^{2}$ and 150 fs, the molecular dynamics is traced in terms of the degree of alignment $\langle \cos ^{2}\theta \rangle$ (Fig. 2a), which exhibits the same dynamics as shown in Fig. 1a. The second two-color pulse can effectively orient the molecules in the well-aligned status because the orientation potential itself is typically two orders of magnitude smaller than the alignment potential^42^. Therefore, the even or odd states can be oriented selectively by controlling the delay of the two-color pulse. As shown in Fig. 2c, the even–J states are oriented more strongly by shooting the two-color pulse at $t = t_{\text {2}}$, where the even–J states are aligned and the odd–J states are anti-aligned. Therefore, the net degree of orientation is dramatically enhanced and the $<\cos \theta>$ reaches 0.194 and 0.156 at $t = t_{\text {a}}$ and $t = t_{\text {b}}$, respectively. This two pulse technique for enhancing the molecular orientation has been reported theoretically^31,32^ and experimentally^33–35^

On the other hand, by irradiating the two-color pulse, the preformed well-aligned status can be remarkably disturbed, as can be confirmed in Fig. 2b (comparison between dashed lines and solid lines), which has not been pointed out in the previous studies. As a result, while the two-color pulse generates the orientation, the alignment is disturbed, and the modulations of the alignment and orientation are not temporally synchronized. Considering the alignment, if a researcher wants to enhance the alignment by shooting dual pulses, the second pulse must arrives when the degree of alignment along the polarization starts to convert from poor alignment to good alignment so that the torque impacted by the second pulse on the molecular axis is maximized^43^. In the orientation methods, however, the two-color pulse counteracts the alignment method. That is, the second pulse orients the even-J states selectively, and at the same time, it also partially cancels and disturbs the preformed alignment of the even–J states. The high degree of orientation, $\langle \cos \theta \rangle$: 0.1–0.5, means the plus-minus inversion asymmetry is remarkable, which does not necessarily mean that the molecules are well confined in the axis. As can be seen in Fig. 2d, e, the rotational wavepacket of the oriented even–J states have worse alignment compared to the alignment at $t=t_{2}$ (see the inset in Fig. 2a). Therefore, under the all collinearly-polarized condition, it is unclear if the oriented molecular sample is well confined in the axis of the laser polarization.

### Two pulse orientation (one-color pulse and crossed-polarized two-color pulse)

Figure 3a, b show the time evolution of the degrees of alignment and orientation after the second crossed-polarized pulse irradiation at $t=t_{2}$. The alignment dynamics launched by the first pulse can be preserved (or even enhanced) if a crossed-polarized two-color pulse is used. The two-color pulse itself does not introduce a significant alignment potential because the two wavelength components have equal intensity. The two-color pulse forces the molecular axis to be confined in the *ZX*-plane, which is equivalent to the anti-alignment against the *Y*-axis. At the same time, the two-color pulse can generate the orientation of the molecule in the *Z*-direction. We note that the orientation potential in the *Z*-direction, which is much weaker than the alignment potential, can play a role only when the molecules are aligned in the *Z*-axis^39^. Therefore, without destroying the preformed alignment in the *Z*-axis, the orientation can be generated in the temporally synchronized way with respect to the alignment. Furthermore, the odd–J states have much smaller degrees or alignment and negligible degrees of orientation in the *Z*-direction. As a consequence, at $t=t_{\text {c}}$ the maximum net degree of orientation $<\cos \theta>$: 0.205 is achieved, showing a similar value compared to the linear polarization method. As can be seen in Fig. 3c, the very well aligned and oriented molecular sample can be obtained from the even–J states. This strategy using the crossed field, therefore, has a notable impact on the field-free orientation technique.

### Degree of orientation of molecules probed by linearly polarized laser

The two different polarization configurations provide similar molecular orientation in terms of the achieved maximum macroscopic degree of orientation $<\cos \theta>$. However, in reality $<\cos \theta>$ itself or even together with $<\cos ^{2}\theta>$ does not provide full information on the molecular rotational distribution. In fact, the rotational wavefunctions shown in Figs. 2d, e, and 3c have completely different shapes; yet, they have the similar values of $<\cos \theta>$. In many experiments, linearly polarized laser is used to probe the statuses of molecules via nonlinear processes triggered by tunneling such as Coulomb explosion and high-order harmonics. The tunneling probability very strongly depends on the angle between the laser polarization and a molecular axis; molecules have maximized tunneling probability in the specific direction. We have examined the up-down asymmetry of tunneling probability, $(W_{{\uparrow }})/(W_{{\uparrow }}+W_{{\downarrow }})$, for a molecular ensemble shown in Figs. 2d, e, and 3c. The angle-averaged probabilities are given by

7 $$\begin{aligned} W_{{\uparrow }} = \frac{1}{2\pi }\int _{0}^{2\pi }d\phi \int _{0}^{\pi /2}d\theta \vert Y(\theta ,\phi )\vert ^{2} \Gamma (\theta )\sin \theta , ~~~~~~W_{{\downarrow }} = \frac{1}{2\pi } \int _{0}^{2\pi }d\phi \int _{\pi /2}^{\pi }d\theta \vert Y(\theta ,\phi )\vert ^{2} \Gamma (\theta )\sin \theta , \end{aligned}$$

where $\vert Y(\theta ,\phi )\vert ^{2}$ and $\Gamma (\theta )$ are the angular distribution of molecules and the angle-dependent ionization probability. Taking an analytic expression of the angle-dependent $\Gamma (\theta )$ for OCS molecule from Ref.^44^, we evalulate the up-down asymmetry of the ionization probability. For the wavefunctions shown in Figs. 2d, e, and 3c, the asymmetries are calculated to be 0.845, 0.815 and 0.862, respectively. In terms of the total ionization probability of $W_{{\uparrow }}+W_{{\downarrow }}$, wavefunction shown in Fig. 3c has approximately 1.5 times higher value than the others. This illustrates that in the crossed field configuration, due to the narrow angular distribution of the molecular ensemble in the *Z*-axis, the effective up-down asymmetry and the anisotropic response of the molecular sample can be more enhanced, which will depend on the target molecule.

## Conclusion

A high degree of orientation (the plus-minus inversion asymmetry) does not necessarily ensure that the molecules are tightly aligned. In the collinear polarization configuration, even–J states are well oriented in the *Z*-direction, and the odd–J states are less oriented in the opposite direction at $t = t_{\text {b}}$. On the other hand, with the crossed polarization, the even–J and odd–J states are tightly aligned in the *Z*- and *X*- axes respectively, and only the even–J states are well oriented in the *Z*-direction. Most linear molecules have anisotropic polarizability and ionization probability, which are maximized in the molecular axis. Therefore, the X-aligned odd—J states would not be probed by a linearly polarized laser pulse and most of the observation can be made from the tightly aligned and oriented even—J states. This study confirms the advantages of the crossed-polarization technique over the collinearly-polarization technique: temporally synchronized higher degree of orientation and alignment. Because the maximum laser intensity for the molecular orientation is limited by the ionization threshold of the target molecule, a much higher laser intensity can be used because the instantaneous field strength $\vert E(t)\vert$ is reduced by a factor of $\sqrt{2}$ in the crossed polarization configuration. From the points of general quantum control, the crossed-field configuration is capable to provide a route to achieve a desired target quantum state. For example, the two-color field with crossed polarization can generalize the previous reported unidirectional molecular rotation^45,46^, to the unidirectional rotation of the oriented molecules. The experimental studies on the molecular structure and many molecular-anisotropy-based phenomena will benefit considerably from this method.
